# CRISPR/Cas9 Mutant Rice *Ospmei12* Involved in Growth, Cell Wall Development, and Response to Phytohormone and Heavy Metal Stress

**DOI:** 10.3390/ijms232416082

**Published:** 2022-12-16

**Authors:** Zhaoyang Li, Muhammad Junaid Rao, Jiaying Li, Yanting Wang, Peng Chen, Hua Yu, Chongjian Ma, Lingqiang Wang

**Affiliations:** 1 College of Plant Science and Technology, Biomass and Bioenergy Research Center, Huazhong Agricultural University, Wuhan 430070, China; 2 State Key Laboratory for Conservation and Utilization of Subtropical Agro-Bioresources, College of Agriculture, Guangxi University, Nanning 530004, China; 3 Guangxi Key Laboratory of Sugarcane Biology, Guangxi University, Nanning 530004, China; 4 Henry Fok School of Biology and Agriculture, Shaoguan University, Shaoguan 512005, China

**Keywords:** *Oryza sativa* L., pectin methylesterase inhibitor, cell wall, plant growth regulators, Cd stress

## Abstract

Pectin is one of the constituents of the cell wall, distributed in the primary cell wall and middle lamella, affecting the rheological properties and the cell wall stickiness. Pectin methylesterase (PME) and pectin methylesterase inhibitor (PMEI) are the most important factors for modifying methyl esterification. In this study, 45 *PMEI* genes from rice (*Oryza sativa* L.) were screened by bioinformatics tools, and their structure, motifs, cis-acting elements in the promoter region, chromosomal distribution, gene duplication, and phylogenetic relationship were analyzed. Furthermore, CRISPR/Cas9 was used to edit the *OsPMEI12* (*LOC_Os*03G01020) and two mutant *pmei12* lines were obtained to explore the functions of *OsPMEI* in plant growth and development, and under cadmium (Cd) stress. Compared to wild type (WT) Nipponbare, the second inverted internodes of the mutant plants shortened significantly, resulting in the reduction in plant height at mature stage. The seed setting rate, and fresh and dry weights of the mutants were also decreased in mutant plants. In addition, the pectin methylation of *pmei12* lines is decreased as expected, and the pectin content of the cell wall increased at both seedling and maturity stages; however, the cellulose and hemicellulose increased only at seedling stage. Interestingly, the growth of the *pmei12* lines was better than the WT in both normal conditions and under two phytohormone (GA_3_ and NAA) treatments at seedling stage. Under Cd stress, the fresh and dry weights were increased in *pmei12* lines. These results indicated that *OsPMEI12* was involved in the regulation of methyl esterification during growth, affected cell wall composition and agronomic traits, and might play an important role in responses to phytohormones and stress.

## 1. Introduction

Cell walls are complicated structures that play significant roles in plant growth and development and respond to different stresses. A plant’s primary cell wall mainly consists of hemicellulose and pectin, as well as cellulose microfibrils, along with proteins and aromatic substances [1]. Pectin is composed of homogalacturonans (HG), rhamnogalacturonans I and II (RGI, RGII), and the C-6 carboxylic acid groups of galacturonic acid (GalUA), a component of HG which can be methyl-esterified by methyltransferase [2]. Pectin is a major polysaccharide of plant cell walls and pectin methylesterase inhibitors (PMEI) have inhibitory effects on a variety of plant pectin methylesterase (PME) [3,4]. Plant PMEI is classified by protein sequence and belongs to the Hidden Markov Model PF04043 [5]. PMEI binds to the active site of PME, rendering the substrate unable to bind to the active site of PME [6]. The stability of the combination of PMEI and PME is strongly affected by pH. A study on the binding kinetics between PME and PMEI in tomatoes showed that the dissociation constant was 53 nM at pH 7; however, at 6 pH, the value drops 10 times to 5 nM [7]. PMEI in plants not only has an inhibitory effect on the PME of their species but also has activity on the PME of other plants [3]. Through the study of tomato PMEI, it was found that tomato PMEI had no inhibitory effect on the PME of bacteria (*E. chrysanthemi*) [8]. According to modeling and fluorescence localization studies, the active sites of PME are located in the cracks of the protein, while the cracks in the active region of PME in fungi are deeper than those in plants, and the plant PMEI cannot bind to the active region to inhibit its activity; in addition, the PME sequence in fungi has no specific conservative sites [8,9].

PMEI mediates the change in PME activity, leading to the change in plant methyl ester, which affects the development of plant cell walls, pollen tube elongation, root development, and stress resistance [10]. In rice, with overexpression of *OsPMEI28*, the plant height becomes shorter than the wild type [11]. After overexpression of *AtPMEI5* (activates the brassinolide ‘BR’ signaling pathway), the stems and leaves of transgenic lines showed an irregular distorted abnormal phenotype, and the pods were deformed [12,13]. In addition to being a component of the cell wall, pectin combines with the extracellular domain of the cell wall receptor kinase (WAK) to regulate WAK activity, thereby further regulating the response of plant hormones [14,15,16]. The change in pectin methylation usually affects the change in the plant hormone (BR) signaling pathway. The increase in pectin methylation affects the combination with WAK, thus affecting the signal pathway, leading to the inhibition of plant growth [12,16,17].

The abnormal development of pollen and anther is known as genetic male sterility (GMS). As shown in previous studies, *OsPMEI24* (*LOC_Os06g49760*) may participate in cytoplasmic male sterility (CMS), a type of GMS caused by cytoplasm. [18,19]. GAs are involved in the development of male organs in most plants [20]. There was a significant difference in plant hormone content between CMS and maintainer lines of pepper, with high IAA and ABA content, and lower ZR_5_ and GA_3_ content, while IAA was involved in vascular bundle differentiation [21]. Through transcriptome analysis of the *AtPME3* mutant, it was found that the expression of gibberellin-related genes and some receptor kinase genes decreased significantly [13]. A variety of plant hormones participate in CMS. The expression of *CaPMEI1* is regulated by different signal molecules such as salicylic acid, jasmonic acid, ethylene, hydrogen peroxide, etc. [22]. *AtPMEIs* were differentially expressed in the treatment of abscisic acid, gibberellic acid, auxin, and methyl jasmonate in *Arabidopsis* [23]. In maize anthers, the expression of 35 *ZmPME*/*ZmPMEI* genes was higher than in other tissues [24].

The negatively charged carboxyl group of pectin has a strong affinity to positively charged heavy metal cadmium, and the content of pectinuronic acid is positively correlated with the cadmium accumulation in stems [25,26,27]. In aluminum (Al) stress culture of tobacco and maize, it was found that 72~82% of Al in tobacco was combined with pectin, the Al content in pectin at the root tip of the maize plant was higher, and the absorption of heavy metal Al was positively correlated with pectin content [28,29,30]. The results showed that 60% of copper ions were bound to the HG of cell wall pectin after the vines were cultured under copper stress [31]. Through a study on Cd stress of flax and carnation roots, it was found that the content of low methyl pectin in the cell wall was positively related to the adsorption of heavy metal Cd, and the regulation of PME and PMEI on the methylation of pectin greatly affected the adsorption of heavy metals by plants [26,27].

The content of pectin in the primary cell wall and the intercellular layer between the cell wall is relatively high, and different modification methods endow it with different effects. The methylation modification plays an important role in the process of plant cell wall extension and stress response. Among them, PMEI plays an important role in affecting pectin, and methyl esterification modification plays an important role. There is much research on the function of *PMEI* in *Arabidopsis*, but the research on the effect of *PMEI* on plant growth in rice is still insufficient.

CRISPR/Cas9 gene editing technology has been widely used for studying agronomic traits and metabolic pathways and. improving the quality and quantity of crop plants [32,33]. In this study, the OsPMEI protein members in rice were phylogenetically analyzed and, the motif composition and gene structures of the *PMEI* genes were investigated. For one *OsPMEI12* gene, the vector was constructed by CRISPR/Cas9 gene editing technology, and two types of gene-edited lines were obtained through the method of genetic transformation. The agronomic traits of the gene-edited lines were investigated, their effects on the growth of seedlings were observed, and cell wall components, as well as a phytohormone and heavy metal treatments, were determined. This study will enhance our understanding of the effect of *OsPMEI* on rice growth and cell wall synthesis.

## 2. Results

### 2.1. The Classification, Chromosomal Distribution, and Gene Duplication of OsPMEI Gene Family

**Figure 1:**
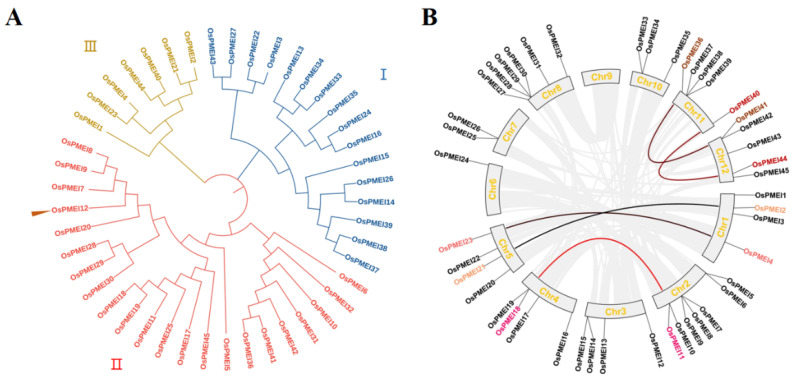
Phylogenetic relationship (**A**), and chromosomal distribution and duplication events (**B**) of the *OsPMEI* family in rice.

The MEGA7 software was used to construct a phylogenetic tree and the 45 OsPMEIs proteins could be divided into three groups I, II, and III, each containing 16, 22, and 7 members, respectively. OsPMEI12 belongs to group II (Figure 1A). The chromosomal distribution and gene duplication the of the *OsPMEI* members were visualized by TBtools (Figure 1B). The Chr2 was the most distributed with seven *OsPMEI* genes, Chr6 was the least distributed with only one *OsPMEI* gene, Chr9 had no *OsPMEI* gene, and other chromosomes had two to six *OsPMEI* genes. *OsPMEI12* is located on Chr 3, where five pairs of gene duplication (*OsPMEI36*/*OsPMEI41*, *OsPMEI40*/*OsPMEI44*, *OsPMEI2*/*OsPMEI21*, *OsPMEI4*/*OsPMEI23*, and *OsPMEI11*/*OsPMEI18*) were found.

### 2.2. Gene Structure and Cis-Acting Elements in the Promoter Regions of OsPMEI Family

**Figure 2:**
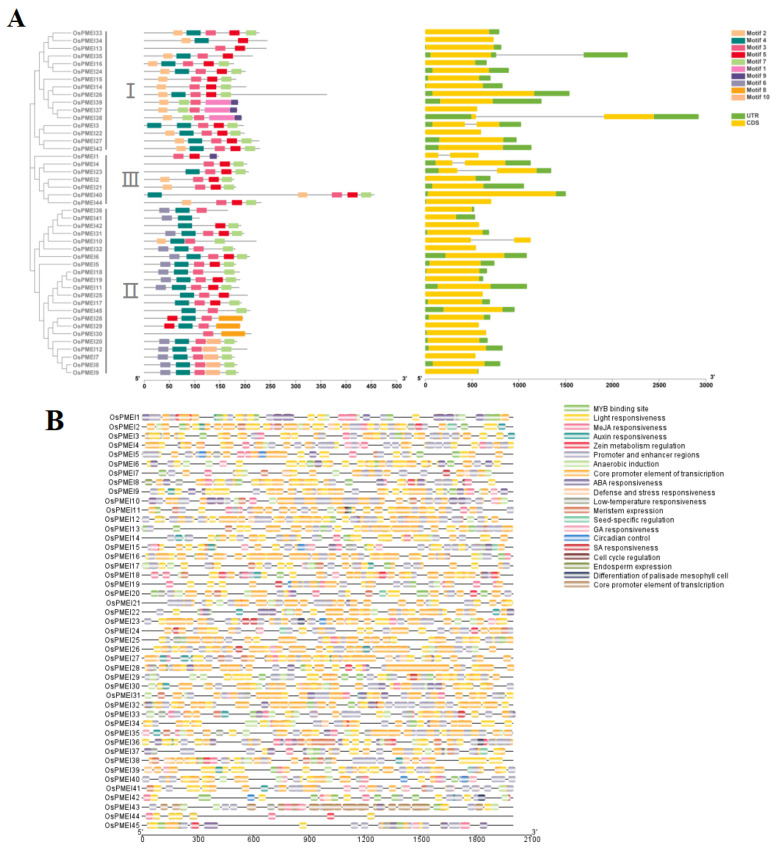

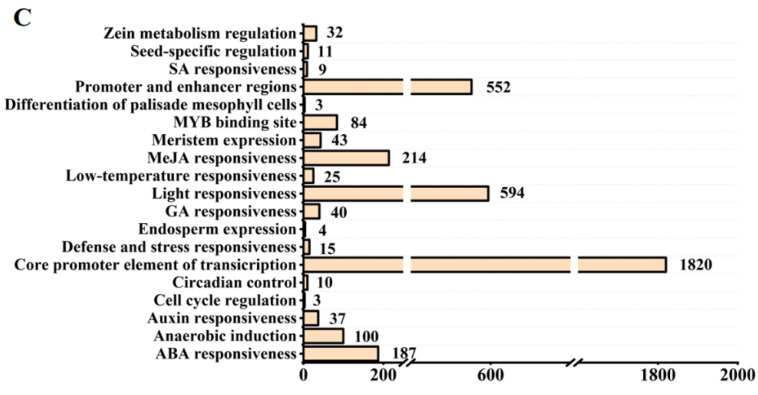
Gene structure and cis-elements of *OsPMEI* family members in rice. (**A**) Motifs and gene structures of *OsPMEIs*; (**B**) Cis-elements in the promoter regions of *OsPMEIs*; (**C**) Statistics of cis-elements of *OsPMEIs*.

TBtool was used to extract the gene structure information of *OsPMEIs* and the motif composition of *OsPMEI* genes were predicted by MEME (Figure 2). It was found that six genes (*OsPMEI1*, *OsPMEI4*, *OsPMEI10*, *OsPMEI13*, *OsPMEI23*, and *OsPMEI38*) each have two exons, while the others have only one exon. However, the gene structure and the motif composition were similar among the members. Most *OsPMEI* gene members contain five common motifs (motif 2–5, and motif 7), while motif 6 was only shared by the members in group II.

We predicted the cis-acting elements on the promoter regions of the *OsPMEI* gene family (Figure 2B). The *OsPMEI* gene family contains the most transcriptional core promoter elements, followed by light-responsive elements. Among hormone-responsive elements, methyl jasmonate-responsive cis-elements have a maximum of 214, followed by 187 abscisic acid-responsive elements. There are 37, 40, and 9 elements for responses of auxin, gibberellin, and salicylic acid, respectively. Among the total 140 stress response elements, anaerobic inducible cis-elements is the most abundant type with the number 100. In addition, cis-elements also include cis-acting elements that regulate plant growth and metabolism, including circadian regulatory elements, meristem expression elements, palisade mesophyll cell differentiation elements, cell cycle regulatory elements, endosperm expression elements, seed-specific regulatory elements, and zein metabolic regulatory element (Figure 2B,C). Among them, the *OsPMEI12* promoter has five abscisic acid response elements, four methyl cyanate re-sponse cis-elements, two meristem expression elements, one auxin response element, one anaerobic inducible element, and one zein metabolic regulatory element (Figure 2C).

### 2.3. Subcellular Localization of the OsPMEI12 Protein

**Figure 3:**
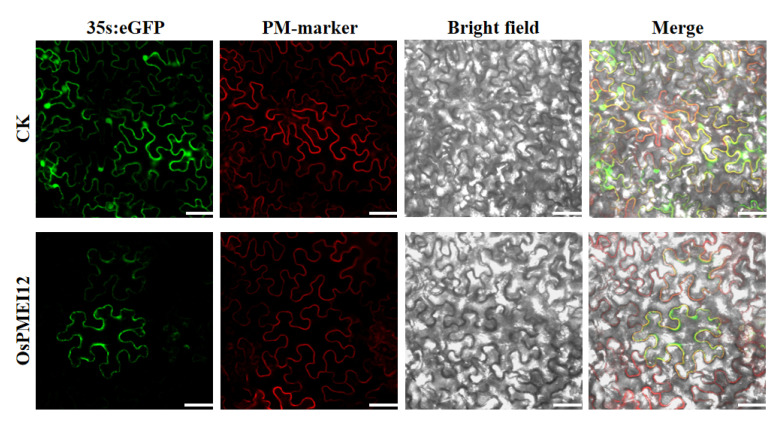
Subcellular localization of OsPMEI12 overserved with confocal microscopy. CK: PD1301s-eGFP no-load with plasma membrane marker transformation; OsPMEI12: pD1301s-eGFP-OsPMEI12 is co-transformed with PM-marker. Scale bars: 30 µm.

The subcellular localization of a protein is one important aspect of its function. We fused the *OsPMEI12* gene with green fluorescent protein (GFP) to construct a recombinant vector. Then, the vector was co-transformed with a plasma membrane marker (PM-marker) in pant *Nicotiana benthamian*. The subcellular localization experiment showed that the OsPMEI12 protein expressed in the plasma membrane, co-localizing with the PM-marker (Figure 3).

### 2.4. The Agronomic Traits and Vascular Bundles of OsPMEI12-Edited Lines

**Figure 4:**
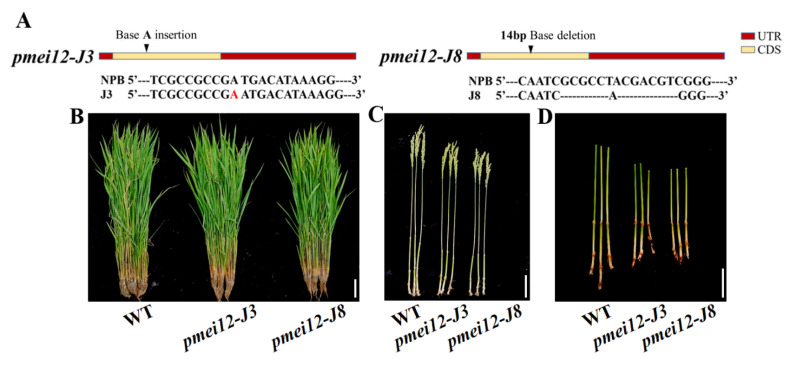

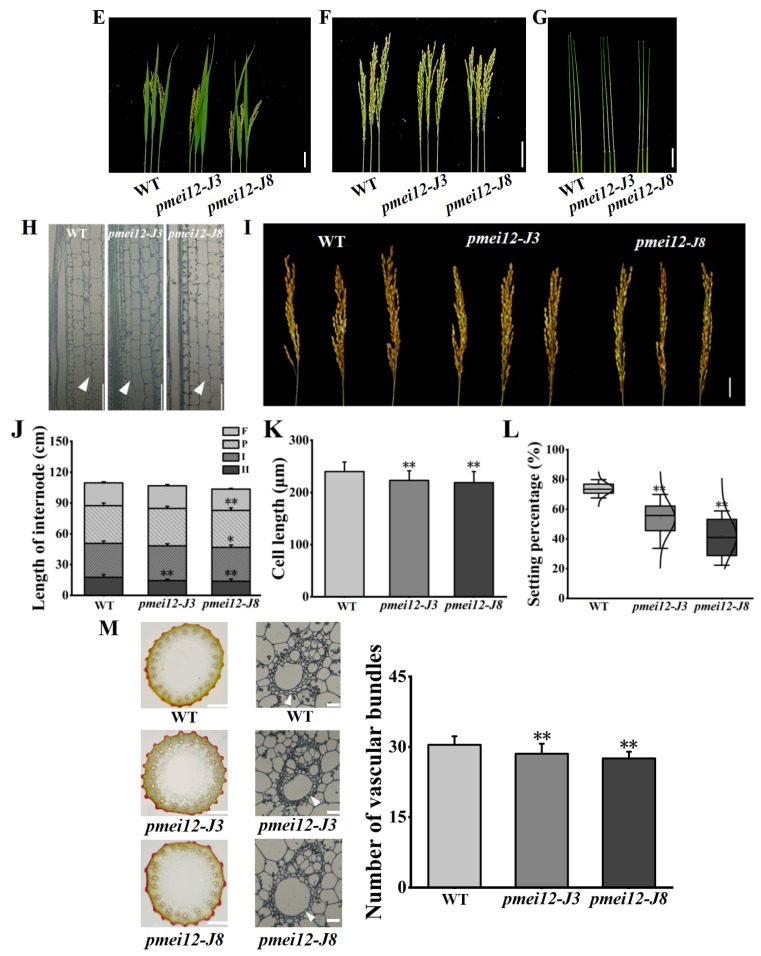
Agronomic traits and vascular bundles of *pmei12* lines and wild type (WT). (**A**) Mutant information; (**B**–**G**,**I**) Agronomic traits of *pmei12s* and WT; (**H**) Section of the second internode; (**J**) L of flag leaf, panicle, and internode of WT and *pmei12* lines (F: flag leaf; P: panicle length; I: the first internode; II, the second internode); (**K**) Cell length of the second internode (µm); (**L**) Setting percentage (%). (**M**) Vascular bundles of *pmei12* lines under compound microscope (500 µm, 100 µm) and triangles indicated the vascular bundle sheath. * and ** indicated the significant differences between *pmei12* lines and WT by *t*-test at *p* < 0.05 and 0.01, respectively. Scale bars: (**B**,**D**) 10 cm; (**C**,**E**–**G**,**I**) 5 cm; (**H**) 100 µm (n = 30).

Two independently transgenic lines (*Ospmi12-J3* and *Ospmei12-J8*) were obtained by using CRISPR/Cas9 gene editing technology. The *Ospmi12-J3* was the homozygous mutant with one base pair ‘A’ insertion in the target site of the exon, while *Ospmei12-J8* was the homozygous mutant with 14 base pairs deletion in the target site of the exon (Figure 4A). Agronomic traits were evaluated in two *pmei12* lines compared with wild type (WT) Nipponbare (Table 1; Figure 4). At mature stage, the plant height of the edited lines *pmei12-J3* and *pmei12-J8* were significantly decreased by 6.3% and 9.0%, respectively (Table 1). However, there was no significant difference in the tiller number, effective panicle number, and 1000-grain weight of *pmei12* lines as compared with WT.

It was found that the decrease in plant heights of two mutant lines at mature stage was mainly caused by the decreased length of second inverted internodes of *pmei12-J3* and *pmei12-J8* culms (by 18.4% and 21.9%, respectively) compared to the WT (Figure 4). We noticed that the length of panicle and the first inverted internode of *pmei12-J8* was also significantly reduced (Figure 4F,G,J). The cell length of the second inverted internodes of two *pmei12* lines was significantly reduced, however, the cell size remained unchanged (Figure 4D,H,J,K). At grain-filling stage, the lower seed setting rate (by 26.21% and 42.8%) and increased black husk rate of *pmei12-J3* and *pmei12-J8* were observed (Figure 4I). In addition, it was found that the number of vascular bundles in the second inverted internode of the two *Ospmei12* lines was significantly lower than that of WT (Figure 4M). The vascular bundle sheath of two *pmei12* lines is thinner than that of WT.

### 2.5. OsPMEI12 Affects Anther Growth and Pollen Fertility

**Figure 5:**
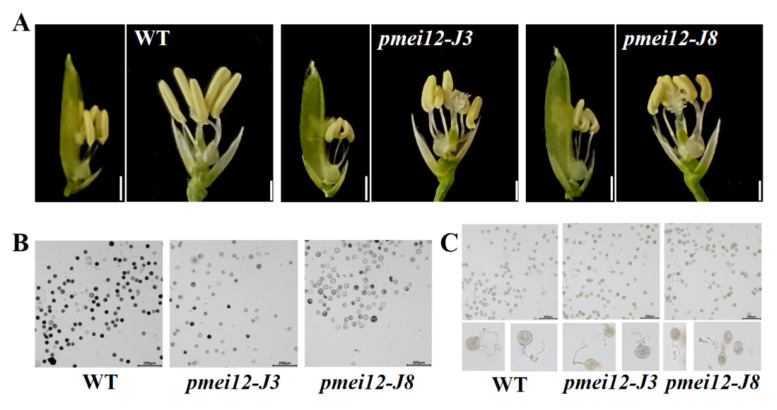

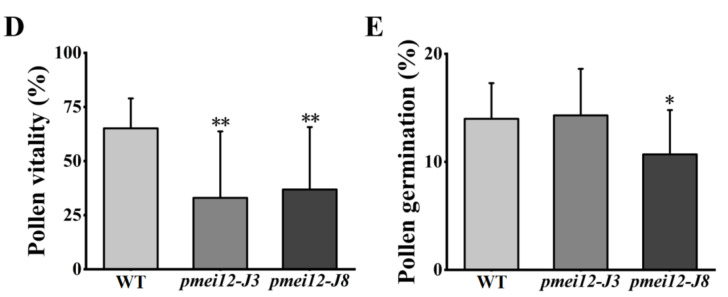
Pollen fertility of WT and *pmei12* lines. (**A**) Anthers of the spike in middle; (**B**) Pollen activity; (**C**) Pollen tube growth of rice; (**D**) Pollen vitality (**E**) Pollen germination. * and ** indicate the significant difference between *pmei12* lines and WT by *t*-test at *p* < 0.05 and 0.01, respectively. Scale bars: (**A**) 2 mm; (**B**,**C**) 200 µm (n = 50).

To explore the reasons for the reduced seed setting rate of the two *pmei12* lines, we observed the anthers of the WT and *pmei12* lines (Figure 5A–C). Compared with WT, the anthers of *pmei12* were shorter in length and irregularly curved (Figure 5A). According to the observation of pollen activity, it was found that the pollen activity of the *pmei12* lines was lower than that of WT, mainly due to the total pollen abortion of the entire anther in the *pmei12* lines (Figure 5B). Previously, it was reported that the exogenous addition of PME could inhibit pollen tube elongation [34]. The results of the pollen tube germination experiment showed that the pollen tube germination of *pmei12-J8* was inhibited, and there was no significant difference between *pmei12-J3* and WT (Figure 5C,E). After observing the pollen tube length, pollen tube length inhibition was not found; *OsPMEI12* may have less effect on pollen tube elongation.

### 2.6. OsPMEI12-Edited Lines Showed Fast Growth and Altered Responses to Phytohormone at Seedling Stage

**Figure 6:**
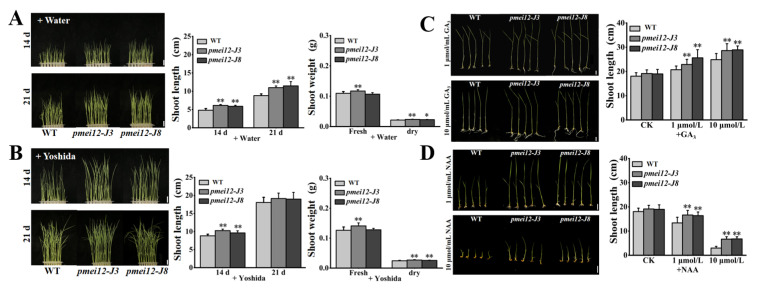
The seedling of *pmei12* lines and WT under Yoshida nutrient solution and under phytohormone treatments. (**A**) Seedlings of WT and *pmei12* lines + Water; (**B**) Seedlings of WT and *pmei12* lines + Yoshida; (**C**) Seedling growth with GA_3_ (1 μmol/L and 10 μmol/L); (**D**) Seedling growth with NAA (1 μmol/L and 10 μmol/L). * and ** indicate the significant differences between *pmei12* lines and WT by *t*-test at *p* < 0.05 and 0.01, respectively. Scale bars: (**A**,**B**) 2 cm, (n = 30); (**C**,**D**) 1 cm, (n = 30).

It was found that before the three-leaf stage, the height of two *pmei12* lines was higher than that of WT (Figure 6). To explore whether it was caused by the difference in seed storage or not, we compared the seedlings’ growth with and without the addition of Yoshida. After 14 days of treatment, the seedling height of the WT was lower than that of the *pmei12* lines (Figure 6A,B). The growth period of the shoot in WT was also slower than that of the *pmei12* lines. After 21 days of growth without nutrient solution from germination, the seedling height of *pmei12* lines was higher (Figure 6A,B), but there was no significant difference in the height of seedlings grown under nutrient solution. The fresh weight of *pmei12-J3* was significantly higher than WT, both with and without nutrient solution, and the dry weight of both two mutant lines significantly increased compared to WT.

Since in *pmei12* lines, whose anthers are smaller, vascular bundle differentiation was different and seedling growth was accelerated, we speculate that it may be caused by different responses to plant hormones. In addition, the upstream of *OsPMEI12* gene contains auxin and GAs response elements. Therefore, we applied exogenous GA_3_ and NAA (1 μmol/L and 10 μmol/L) to 7-day-old seedlings after germination with Yoshida nutrient solution to evaluate the growth of *pmei12* lines (Figure 6A,B). After 14 days of treatment with GA3, all seedlings were higher than the control group, however, the seedling height of *pmei12* lines was much higher than that of WT (Figure 6C). The growth period of WT seedlings is relatively slow. After adding NAA for 14 days, the growth of the seedlings was all slower than the control group. The growth of the seedlings was greatly restricted under the treatment of high concentrations of NAA, especially the WT (Figure 6D). However, the mutant seedling height was significantly higher than that of WT under both concentration treatments, indicating the better performance of the *pmei12* lines (Figure 6C,D).

### 2.7. OsPMEI12 Affects the Biosynthesis of Cell Wall Components

**Figure 7:**
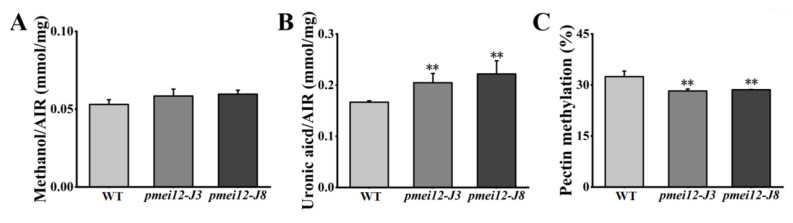

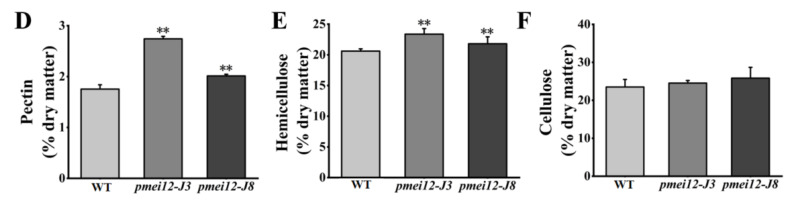
Cell wall composition of *pmei12* lines and WT at seedling stage. (**A**) Methanol (AIR); (**B**) Uronic acid (AIR); (**C**) Pectin methylation (%); (**D**) Pectin content dry matter; (**E**) Hemicellulose content dry matter; (**F**) Cellulose content dry matter. ** indicate the significant differences between *pmei12* lines and WT by *t*-test at *p* < 0.01 (n = 3).

To explore the influence of *OsPMEI12* on pectin methyl-esterification and the difference of seedling dry weight, we determined the degree of pectin methyl-esterification by measuring the ratio of uronic acid to methoxy group in unit mass alcohol insoluble substance (AIR). Methoxy content was determined after methanol is released through the AIR saponification reaction [35]. We measured the methylation degree and cell wall component of seedlings after 21 days of growth. There was no significant difference in the methanol content released by WT and *pmei12* lines, but the uronic acid content of *pmei12* lines was significantly increased, leading to a synergetic decrease in the methyl-esterification degree of *pmei12* lines (Figure 7A–C). Additionally, we determined pectin, cellulose, and hemicellulose content in the dry matter [36]. The results showed that the pectin and hemicellulose content in *pmei12* lines increased significantly compared to WT, however, the cellulose content did not change significantly (Figure 7D–F). At mature stage, the content of uronic acid and pectin in *pmei12* lines increased significantly compared to WT (Figure 7B,D). However, there was no significant difference between hemicellulose and cellulose at mature stages (Figure S1). Immunofluorescence observation on the mature stems showed that the uronic acid contents were increased (Figure S2).

### 2.8. OsPMEI12 Is Involved in Cadmium Stress Response

**Figure 8:**
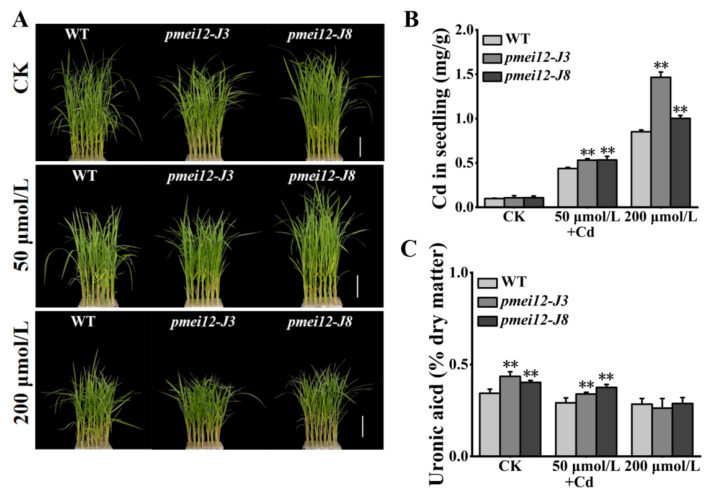

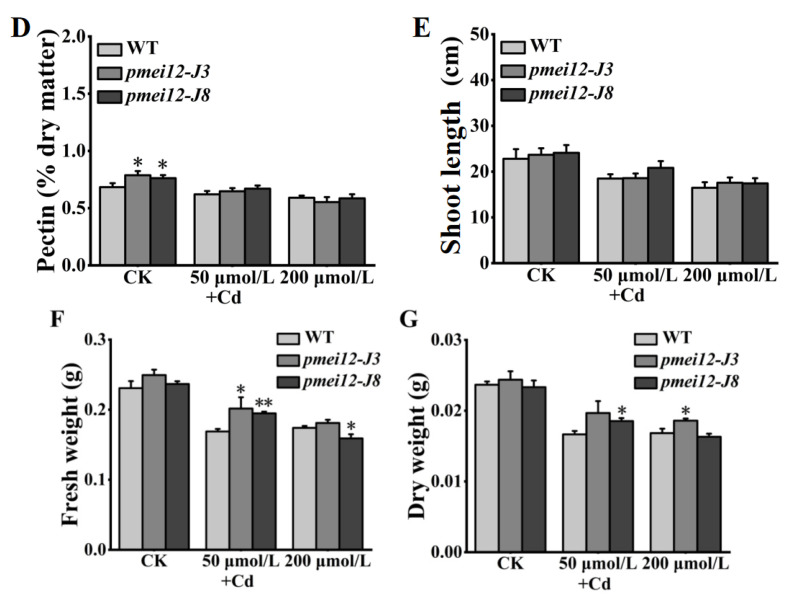
Seedlings treated with heavy metal Cd stress. (**A**) Plants of *pmei12* and WT under Cd treatments; (**B**) Cd content of seedlings (mg/g); (**C**) Uronic acid content in seedlings (dry matter); (**D**) Pectin content in seedlings (dry matter); (**E**) Shoot length (cm); (**F**) Fresh weight of seedlings; (**G**) Dry weight of seedlings. * and ** indicate the significant difference between *pmei12* lines and WT by *t*-test at *p* < 0.05 and 0.01, respectively. Scale bars: (**A**) 5 cm (n = 3).

The change in pectin methyl ester will affect the adsorption of heavy metals [26,27]. We set cadmium (Cd) stress (50 μmol/L and 200 μmol/L) to treat 21-day-old seedlings. After 15 days of treatment, it was found that WT was better than the *pmei12* lines, however, there was no significant difference in appearance (Figure 8A,E). Under Cd treatment, the Cd content of *pmei12* lines was significantly higher than WT, and the Cd adsorption capacity of *pmei-J3* under high treatment increased by 71.97% compared to WT (Figure 8B). Compared with WT, the contents of uronic acid and pectin (dry matter) in *pmei12* lines decreased with the increase in Cd treatment concentration (Figure 8C,D). The dry weight of *pmei12* decreased with the increase in treatment concentration. Under low treatment, the dry weight of WT decreased by 29.58%, and the dry weight of *pmei12* lines decreased by 19.31% and 23.74%, respectively. Under high treatment, the dry weight of WT decreased by 24.56%, and the dry weight of *pmei12* lines decreased by 23.74% and 30.09%, respectively (Figure 8F,G). Additionally, there was no significant difference in soluble sugars and hemicellulose, however, the cellulose increased significantly in *pmei12* lines compared to in WT (Figure S3).

## 3. Discussion

### 3.1. The Dynamic Effects of OsPMEI12 on the Plant Height of Rice

In plants, PMEI family members have crucial roles in growth and development, abiotic and biotic stress, and cell wall component biosynthesis [37]. In *Arabidopsis*, 71 putative PMEI genes have been reported and in rice, 49 putative PMEI genes were identified previously [11,37]. In our study, we screened 45 *PMEI* genes from rice and their gene structure, cis-acting elements in the promoter region, chromosomal distribution, and gene duplication were investigated (Figure 1). Our results showed that the mutation of *OsPMEI12* reduced the second inverted internode of stems at mature stage, leading to a decrease in plant height compared to WT, which was likely caused by the reduced cell number instead of the cell size in the stems of the mutant plants (Figure 2 and Figure 3). Interestingly, we found that *pmei12s* plants grew faster at seedling stage, despite the mutant plants being slightly shorter compared with WT at mature stage. The fast growth phenotype of *pmei12s* plants can be reinforced by the fact that, under the treatments of Yoshida, GA3, and NAA, the mutant lines grew consistently better than the WT. When we measured the cell wall components of the plants, we found that, at seedling stage, the *pmei12* lines had significantly increased contents of uronic acid, pectin, and hemicellulose compared to WT (Figure 6B–D), however, at mature stage, the contents of hemicellulose and cellulose remained unchanged (Figure 7 and Figure S1). In addition, we analyzed OsPMEI gene expression through the GEO database and found that *OsPMEI12* was significantly higher than other *OsPMEIs* at three-leaf stage, whereas, at other stages, this dominant expression of *OsPMEI12* was not observed. We also noticed that, in a previous study, the overexpression of rice *OsPMEI28* exhibited dramatically dwarf phenotype, significantly reduced plant height, and increased pectin methyl-esterification [11]. They did not observe any distinct phenotypes during early developmental stages, however, OX-OsPMEI28 plants had significantly reduced plant height compared to WT one month later and was manifested at maturity [11]. In *Arabidopsis*, overexpression of *AtPMEI5* caused significantly twisted stems, most noticeably around points on the stem where leaves or inflorescences failed to separate from the main stem at heading stage [38]. These results clearly indicated the dynamic growth pattern of the *PMEI*-modified lines, and the alteration in cell wall components and in gene expression pattern potentially underlying the aberrant growth phenotypes.

### 3.2. OsPMEI12 Involvement in the Pectin Methyl-Esterification and the Contents of the Pectin and Hemicellulose

**Figure 9:**
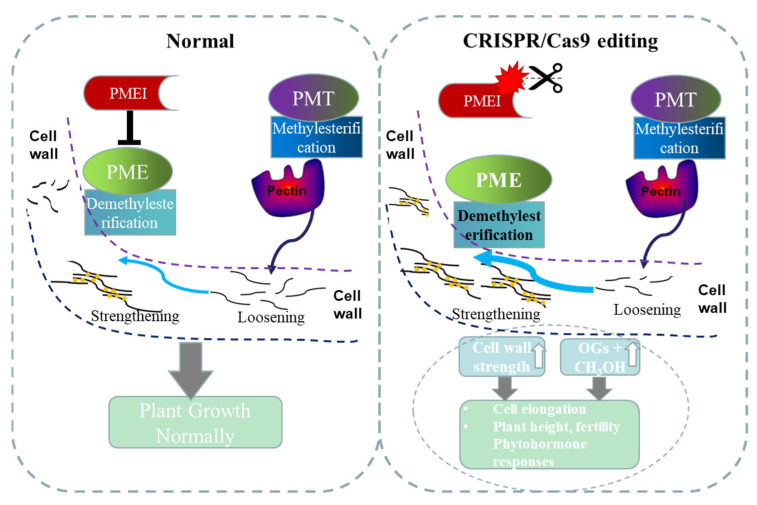
A model was proposed to elucidate that perturbation of OsPMEI and OsPME interaction results in the increased demethylesterification of pectin and has broader effects on cell wall features, plant growth, and phytohormone responses. PMEI: pectin methylesterase inhibitor; PME: pectin methylesterase; PMT: pectin methyltransferases.

PME and PMEI gene families play an essential role in plant cell wall formation [10]. PME and PMEI families’ studies showed that these genes are largely involved in the biosynthesis of pectin and other cell wall components [10,39]. It has been confirmed that OsPMEI12 can interact with PME and inhibit the activity of PME [5]. In this study, we found that the *pmei12* lines significantly increased the contents of uronic acid, pectin and hemicellulose compared to WT at seedling stage (Figure 7). In addition, the pectin methylation degree of the *pmei12* lines was significantly reduced. At first, we measured the methylation of the extracts per unit of alcohol insoluble substance (AIR) and found just slight changes. We suspected that the lack of significant difference in the content of methanol in alcohol insoluble extracts was caused by the significant increase in the content of uronic acid (Figure 7). Since the methanol is reduced from the methoxy group on the uronic acid [39], we calculated the released methanol per unit of uronic acid. It was found that the methylation was significantly decreased in the mutant lines, as expected. It can be understood that the pectin is methylated by the action of pectin methyltransferases (PMT), resulting in a highly mobile state, whereas the pectin methylesterase (PME) actives the demethylesterification of homogalactruonan. PME activity can be specifically regulated by pectin methylesterase inhibitor (PMEI), which is important for maintaining cell morphology and the stability of the middle layer of the cell wall (Figure 9). In this study, CRISPR/Cas9 technology was used to generate two mutant lines *Ospmei1-J3* and *Ospmei1-J8* with 1bp insertion and 14bp deletion in coding sequences, respectively. The PMEI proteins of two mutant lines were functional disability and failed to interact with PME. The demethylesterification activity of PME increased and the precise balance of the degree of pectin methylesterification was affected. Our data indicated that OsPMEI12 functions as a critical structural modulator by regulating the degree of pectin methylesterification and that an impaired status of pectin methylesterification affects the physiochemical properties of the cell wall components and causes abnormal cell extensibility in rice culm tissues (Figure 9).

### 3.3. OsPMEI12 Is Involved in the Hormone Response and Cadmium Stress

The expression of PMEI was reported to be regulated by hormones such as salicylic acid, jasmonic acid, brassinolide, ethylene, abscisic acid, and by methyl jasmonate and hydrogen peroxide in many plants [11,16,17,22,40,41,42]. A wheat PMEI gene was induced in response to hormones (salicylic acid and jasmonic acids) and by hydrogen peroxide, demonstrating that wheat PMEI has a significant role in defense responses [40]. In *Arabidopsis thaliana*, the cell elongation phenomenon is related to cell wall genes, which are regulated by plant hormones [12,16,17]. In previous studies, it was reported that pectin methylation affects wall-associated kinases (WAK) and thereby alters plant responses to brassinolide [14,16,17]. In rice, the increase in pectin methylation triggers the phosphorylation of OsWAK11, and then inhibits the activity of brassinosteroid (BR)-insensitive protein, thus affecting the BR plant hormone response pathway [16]. The OsWAK11 was affected by the change of pectin methylesterification, and its homologous proteins AtWAK1 and AtWAK2 respond to oligosaccharide [14,16]. In this study, it was found that the *OsPMEI* gene family members have large numbers of hormone cis-acting elements in their promoter regions. In addition, the tissue expression profiles indicated that the *OsPMEI* family is involved in the hormone responses of rice plants. After treatment with GA_3_ and NAA, it was found that the mutant *pmei12* plants were more sensitive to GA_3_ and NAA and grew better than WT, providing direct genetic evidence for the roles of PMEI12 and pectin methylesterification in the responses to plant hormone (Figure 6). In addition, we found that protein OsPMEI12 was located in cell membrane like protein WAK11 [16]. These results together indicated that *OsPMEI12* may be involved in WAK-related plant hormone response pathways. We speculate that *OsPMEI12* has an inhibitory effect on rice seedling growth and is regulated by NAA and GA_3_.

In *Arabidopsis thaliana*, many *PMEI* genes were predicted to be involved in cold, oxidation, drought, salt, heat, and wounding regulation [42]. In rice, a specified set of *PMEI* genes were regulated in response to salt stress, drought stress, anaerobic conditions, and low temperature [11]. Previously, it was revealed that the increase in uronic acid and pectin in rice can improve the adsorption of heavy metal cadmium [25]. In this study, after cadmium (Cd) treatment, the adsorption of Cd in the *pmei12* lines increased compared to the WT (Figure 8B). The increased content of pectin and the lower pectin methylation in *pmei12* lines might facilitate the adsorption of heavy metal Cd, similar to the results reported in previous studies [26,27]. In addition, under Cd stress, the dry weight of *pmei12* lines was significantly higher than WT (Figure 8G), which are potentially important in removing heavy metal from contaminated tailing wastes and producing more biomass [43].

## 4. Materials and Methods

### 4.1. Experimental Materials

#### 4.1.1. Rice Material

The *Oryza sativa* L. *japonica*, Nipponbare was used as genetic material for developing transgenic rice. The CRISPR/Cas9 editing vector pRGEB32 was donated by Professor Xie Kabin of Huazhong Agricultural University. The constructed CRISPR/Cas9 vector was provided by Mrs. Hu Shiping. The vector was cultured in plant tissue using agrobacterium-mediated transgenic method to obtain rice. TBtool was used for gene structure analysis and MEME software was used to predict the motifs in the protein sequence of OsPMEIs [44,45].

#### 4.1.2. Strains and Vectors

*Escherichia coli* DH5α and Agrobacterium tumefaciens strain EHA105 were preserved by the Material Energy Laboratory of Huazhong Agricultural University. The CRISPR/Cas9 editing vector pRGEB32 was donated by Professor Xie Kabin of Huazhong Agricultural University and the subcellular localization vector pD1301s-GFP was transformed by the Biomass Energy Laboratory according to the overexpression vector pD1301s protocol (Figure 9). The green fluorescent protein gene sequence was added at the back end of the insertion region of pD1301s, to obtain pD1301s-GFP. The pD1301s-eGFP-*OsPMEI12* primers are represented in Table S1.

#### 4.1.3. Experimental Instruments and Reagents

A stereo microscope (Leica S6D; Berlin, Germany), vibratome (VT1000S, Leica; Santa Barbara, Los Angeles, CA, USA), nucleotide concentration analyzer (NanoDrop 2000, Thermo Scientific, Waltham, MA, USA), and spectrophotometer (Eppendorf Biophotometer; Waltham, MA, USA) were used in this study. The details of reagents used in this study are as follows: Taq enzyme, KOD Plus, restriction endonuclease, T4 DNA ligase, and DNA molecular mass marker were purchased from TaKaRa (Dalian, China) company; Blend Taq fidelity enzyme is a product of TOYOBO company; Easy-T_3_ intermediate vector is from Quanzhijin company; target gene fragment recovery, plasmid extraction, and agarose gel recovery kits were purchased from Hangzhou Boyi Technology Co., Ltd., Hangzhou, China; yeast extract and tryptone are Oxid products; cellulose complex enzyme (Ningxia Heshibi Company, Ningxia, China), plant culture medium gel (Casein), phytol oxidase, and anthrone were purchased from Shanghai Sigma-Aldrich Company, Shanghai, China; plant growth regulator, MES-Aladdin, rice culture medium and cell wall assay-related reagents and inorganic reagents were purchased from Shanghai Sinopharm Group Chemical Reagent Co., Ltd., Shanghai, China.

### 4.2. Experimental Method

#### 4.2.1. Bioinformatics Analysis

The full-length protein sequences of PMEI in *Oryza sativa* were aligned by ClustalW. The unrooted phylogenetic trees were constructed using the Neighbor-Joining (NJ) method with the following parameters: p-distance model, pairwise deletion, and 1000 bootstrap replicate by MEGA7 software (http://www.megasoftware.net/, accessed on 10 November 2020). The conserved motifs in the PMEI protein sequences were found using multiple expectation maximization for motif elicitation (MEME) program version 4.0 (http://meme-suite.org/tools/meme (accessed on 14 August 2022) with the following parameters: some repetitions, maximum number of motifs set to 10, optimum motif width set to >6 and <200. The Plant CARE online software (http://bioinformatics.psb.ugent.be/webtools/plantcare/html/, accessed on 16 November 2020) was used to predict the promoter element and function of the *OsPMEI* genes [44,45,46,47].

#### 4.2.2. Construction of CRISPR Vector

The target gene gRNA primers were developed using the CRISPR-PLANT website (http://www.genome.arizona.edu/crispr (accessed on 4 December 2020), and BsaI restriction sites were added at both ends of the primers. The primers were named: *020-1F/R*, *020-2F/R*, *020-3 F/R*, *020-4 F/R*, and *020-5 F/R*. After the primers were designed, they were synthesized in Wuhan Tianyi Huiyuan Biological Co., Ltd., Wuhan, China. First, the gRNA duplex was ligated with the pRGEB32 vector, and the reaction system is as follows: diluted double-stranded gRNA primers 2 μL, pRGEB32 plasmid 5 μL, 2 × T_7_ ligase buffer 10 μL, BSA 2 μL, BsaI enzyme 0.5 μL, T_7_ ligase 0.5 μL, ddH_2_O up to 20 μL; PCR reaction parameters: 37 °C for 5 min followed by 41 cycles, 20 °C for 10 min followed by 41 cycles, and 20 °C for 1 h followed by 1 cycle. After the reaction was completed, it was taken out and placed in a 4 °C refrigerator, and the recovery of target fragments was achieved after T-A cloning and transformation in the DH5α *E. coli* strain. CRISPR vector-positive colonies were screened using PCR identification. The PCR was conducted for the identification of transgenic positive seedlings by using rice genomic DNA. The complete DNA extraction method is described in File S1.

#### 4.2.3. PCR Detection of Transgenic Seedlings

The extracted DNA was preliminarily identified as positive seedlings with hygromycin primers HygF, HygR, and specific primers hSpCas9F and hSpCas9R on the pRGEB32 vector, and the DNA of transgenic seedlings was used as a template for PCR amplification. The PCR product was detected with 1.0% agarose gel, and whether the transgenic rice seedling was positive was judged according to the presence or absence of the amplified fragment.

#### 4.2.4. Gene-Specific Primer Design

On the Rice Genome Annotation Project (http://rice.plantbiology.msu.edu; accessed on 23 March 2021), the TIGR Locus with specific primers that detected the gene of the positive seedling was found, the full-length gDNA sequence of the gene was downloaded, and the gene at the target site design-specific primers at the position of 300 bp before and after was located. The Tm value of the primers was selected at about 58 °C, and they were named as 020-1Test-F/R, 020-2/3/4Test-F, etc.

The amplification of the target fragment containing the target site was amplified by PCR with specific detection primers on the gene. The primer sequences are represented in Supplementary Table S1.

#### 4.2.5. Investigation of Agronomic Traits

The rice plants were grown in the greenhouse of Huazhong Agricultural University (25 °C, light for 16 h). First, the seeds were soaked in water and after germination, they were sown in pots and then transplanted at the 4–5 leaf stage.

#### 4.2.6. Investigation of Agronomic Characters of Rice

The rice material was planted in the experimental field in 2021 with three replicates (having 40 plants in each replicate). We have selected 10 individual rice plants at mature stage to measure plant height, internode length, tiller number, total panicle number, and effective grain weight in thousands of panicles. We used an analytical balance to measure the hundred-grain weight and convert it to thousand-grain weight. For the seed setting rate, 5 panicles of each plants were taken to calculate the ratio number of filled grains/total number of grains per panicle with 10 repetitions.

#### 4.2.7. Determination of Rice Pollen Viability

The rice anthers were collected before the flowering stage of the rice. We used tweezers to peel off the anthers and gently shook the pollen onto the glass slide. We used 1% potassium iodide (KI) to stain and press the slides. After standing for 5 min, we used a fluorescence upright microscope (Olympus BX61; Tokyo, Japan) to observe and take pictures.

#### 4.2.8. Rice Pollen Tube Germination

We selected the panicle samples 1 day before the flowering stage, when the anther length was more than 1/2 of the glume. We added a drop of rice pollen germination solution on the glass slide, peeled off the anthers, and shook the pollen into the germination solution. We incubated at 28 °C for 25 min, observed and took pictures under a fluorescence upright microscope (Olympus BX61; Tokyo, Japan).

#### 4.2.9. Extraction of Cell Wall Polysaccharides

We weighed about 0.1000 g of the sample into a mortar, added an appropriate amount of 0.5 mol/L phosphate buffer with pH = 7.0, ground it to a homogenate, transferred it to a 15 mL centrifuge tube, centrifuged (4000 r/min, 5 min), and collected the supernatant. The pellet was washed twice with 5 mL of phosphate buffer and twice with 5 mL of dH_2_O; the following centrifugation was as above. We added 5 mL of chloroform-methanol (1:1, *v*/*v*) to the precipitate, shook at room temperature (about 25 °C) at 150 r/min for 1 h, centrifuged to remove the supernatant, washed the precipitate once with 5 mL of methanol, washed once with 5 mL of acetone, washed once with 5 mL of distilled water, and removed the supernatant. We added 5 mL of DMSO-H_2_O (9:1, *v*/*v*) to the pellet, shook at room temperature overnight (12 h), and, after centrifugation, collected the supernatant, washed the pellet with 5 mL of DMSO-H_2_O twice, and then used 5 mL of DMSO-H_2_O. Than washed 3 times with 5 mL dH_2_O, and the residue after this step was the crude cell wall. For the ammonium oxalate extraction of pectin, we added 5 mL of 0.5% (*w*/*v*) ammonium oxalate to the precipitate, heated it in boiling water for 1 h, shook it every 10 min during this period, centrifuged, collected the supernatant, and made up the volume. A spectrophotometer was used for taking the reading of each sample and compared their absorbance with pentoses, hexoses, and uronic acids (details are provided in Supplementary File S1) [36].

#### 4.2.10. Determination of Pentose, Hexose, and Uronic Acid by Colorimetry

For the hexose, we took 1 mL hexoses to extract the solution in a glass tube, slowly added 2 mL anthrone sulfate reagent (0.2% *w*/*v*), and incubated in boiling water for 5 min. After this, the solution was cooled in a room temperature water bath and absorbance at 620 nm was measured using a spectrophotometer. We calculated the hexose content using the hexose standard curve: y = 107.85x − 0.2386, R^2^ = 0.9995 (y: unit content of matter, x: absorbance). For the pentose, we took 1 mL hexoses extracting solution in a glass tube, added 134 μL A reagent (6.00 g Orcinolmonohydrate dissolved in 100.0 mL absolute ethanol), and 2 mL B reagent (0.10 g FeCL_3_ dissolved in 100.0 mL 98% HCL), incubated in boiling water for 20 min. After cooling, it was measured at 660 nm, using the pentose standard curve: y = 59.101x − 0.9493, R^2^ = 0.9995. For the uronic acid, we took 1 mL extracting solution in a glass tube, added 5.0 mL of 0.50% sodium tetraborate/98% H_2_SO_4_ solution (*w*/*v*), shook well, and incubated in boiling water for 5 min. After cooling, absorbance A was measured at 520 nm and the sample was recovered. We added 100 μL 0.15% m-hydroxybiphenyl solution/H_2_O (*w*/*v*). After fully mixing, we let it stand for 10 min. Again, we measured the absorbance B at 520 nm, using the uronic acid standard curve: y = 175.45x + 0.4545, R^2^ = 0.998 (x: absorbance B − absorbance A). We calculated the proportion of cell wall components in the dry matter by unit matter content [36].

#### 4.2.11. Preparation of Alcohol Insoluble Residue (AIR)

We weighed about 10 g of fresh seedling sample, ground it with liquid nitrogen, added 40 mL chloroform-methanol (1:1, *v*/*v*), placed it in a shaking table at room temperature for 150 r and treated it for 1 h. After the supernatant was removed by centrifugation, 40 mL of 70% ethanol was added to the supernatant 4 times. Finally, we used 100% acetone to hang it again, and put it into the fume hood to dry [48].

#### 4.2.12. Measurements of Methyl-Esterification

We weighed 0.100 g of AIR, and the pectin extraction method and the uronic acid determination method are the same as Section 4.2.7 and Section 4.2.8. Taking 1 mL of pectin extracting solution and 0.4 mL of 0.5 M NaOH, we incubated at 30 °C for 30 min. After neutralization with 0.2 mL 1.0 M HCL we used 2400 g centrifugation for 10 min to obtain methanol extracting solution. Next, 0.125 mL methanol extracting solution was added to 0.125 mL 20 mM HEPES buffer (pH 7.5). Samples were oxidized in 0.25 mL HEPES buffer containing 0.03 U alcohol oxidase. We added 0.1 mL of acetylacetone color-developing solution, placed in a 60 °C water bath for 15 min, and added 0.25 mL ddH_2_O. After the solution was cooled in a room temperature water bath, absorbance at 412 nm was measured using a spectrophotometer and the methanol standard curve: y = 0.2682x + 0.0149, R^2^ = 0.987. We determined the methyl-esterification degree using the ratio of methanol release amount and uronic acid content in unit AIR [13].

#### 4.2.13. Subcellular Localization Analysis

The CDS region of PMEI12 was cloned into the C-terminal of the PD1301s-eGFP vector and fused with the GFP reporter gene. PD1301s-eGFP-PMEI12 and plasma membrane Marker (CBL1, Mcherry) were transformed into *Nicotiana benthamiana* by Agrobacterium (GV3101), and then cultured in darkness for 1–2 days and cultured in light for 1 day. *Nicotiana benthamiana* leaves were taken and we observed the fluorescence signal using a confocal laser scanning microscope (FV12000MPE, Olympus, Tokyo, Japan); GFP: 500–530 nm for emission and 488 nm for excitation and Mcherry: 600–650 nm for emission and 552 nm for excitation [49].

### 4.3. Rice Hormone Treatment and Determination of Cadmium Content in Rice

For plant hormone treatment, we only added the nutrient solution in the WT samples. For the treated experimental samples, we added nutrient solution and hormones (1 μmol/L and 10 μmol/L NAA; 1 μmol/L and 10 μmol/L GA_3_) for 14 days.

We weighed 0.1000 g of dry sample (accurate to 0.0001 g) in a porcelain crucible, transferred it to a muffle furnace, carbonized at 200 °C for 30 min, ashed at 500 °C for 6–8 h, and then cooled (if the ashing of individual samples was not complete, we added 1 mL of mixed acid and heated on a small fire on an adjustable electric furnace). After the mixed acid was evaporated to dryness, we transferred the mixed acid to a muffle furnace at 500 °C and continued ashing for 1~2 h until the samples were ash (completely digested, grayish-white, or light gray). After cooling, we digested the ash with a nitric acid solution (1%), followed by transferring the solution into a 25 mL volumetric flask. We washed the porcelain crucible three times with nitric acid solution (1%), combined all the washing solutions into the volumetric flask, added a nitric acid solution (1%) to the scale line, and mixed well for later use. At the same time, we used nitric acid solution (1%) for a reagent blank test and used an atomic absorption spectrometer to determine cadmium concentration (cadmium standard sample: 3 ng/mL, matrix modifier: 10 g/mL L-ammonium dihydrogen phosphate).

## 5. Conclusions

In this study, bioinformatics analysis of the *OsPMEI* gene family was performed, including cluster analysis of family members, motif composition, and phylogenetic analysis. The two mutant lines (*pmei12-J3* and *pmei12-J8*) were obtained using CRISPR/Cas9 editing technology. It was found that the plant height was lower than that of WT due to the shortening of the second inverted internodes at mature stage, which might be caused by the decreased cell numbers. Interestingly, the *pmei12* lines showed fast growth and altered responses to phytohormone at seedling stage. The pectin methylation degree of the *pmei12* lines decreased, and the contents of uronic acid and pectin increased significantly compared to WT. In the stress experiment, it was found that the cellulose contents, adsorption of cadmium, and the resistance to cadmium stress of the *pmei12* lines increased significantly compared to WT. However, there was no significant difference in the changes of hemicellulose and soluble sugar under Cd stress. This study revealed that *OsPMEI12* plays an important role in seedling growth and pectin status and is regulated by plant hormones and Cd stress.

## Figures and Tables

**Table 1:** Agronomic traits of *pmei12s* and WT.

	Tiller Number	Total Number of Panicles	Effective Panicles	1000 Grain Weight (g)	Plant Height (cm)
WT	48 ± 12	38 ± 3	23 ± 4	21.23 ± 0.32	72.96 ± 3.29
*pmei12-J3*	48 ± 13	43 ± 15	23 ± 9	21.2 ± 0.043	68.37 ± 2.75 **
*pmei12-J8*	50 ± 13	36 ± 10	22 ± 6	21.2 ± 0.16	67.57 ± 3.29 **

** indicated the significant difference in the traits of *pmei12* lines and WT by *t*-test at *p* < 0.01 (n = 10).

## Data Availability

Not applicable.

## Supplementary Materials

The following supporting information can be downloaded at: https://www.mdpi.com/article/10.3390/ijms232416082/s1.
