# Supplementary material for: CRISPR/Cas9 Mutant Rice Ospmei12 Involved in Growth, Cell Wall Development, and Response to Phytohormone and Heavy Metal Stress
Source: Int J Mol Sci. 2022 Dec 16;23(24):16082. doi: 10.3390/ijms232416082 (PMC9784561; doi:10.3390/ijms232416082)
Supplement: Supplementary file 1 [file ijms-23-16082-s001.zip › File S1.pdf]

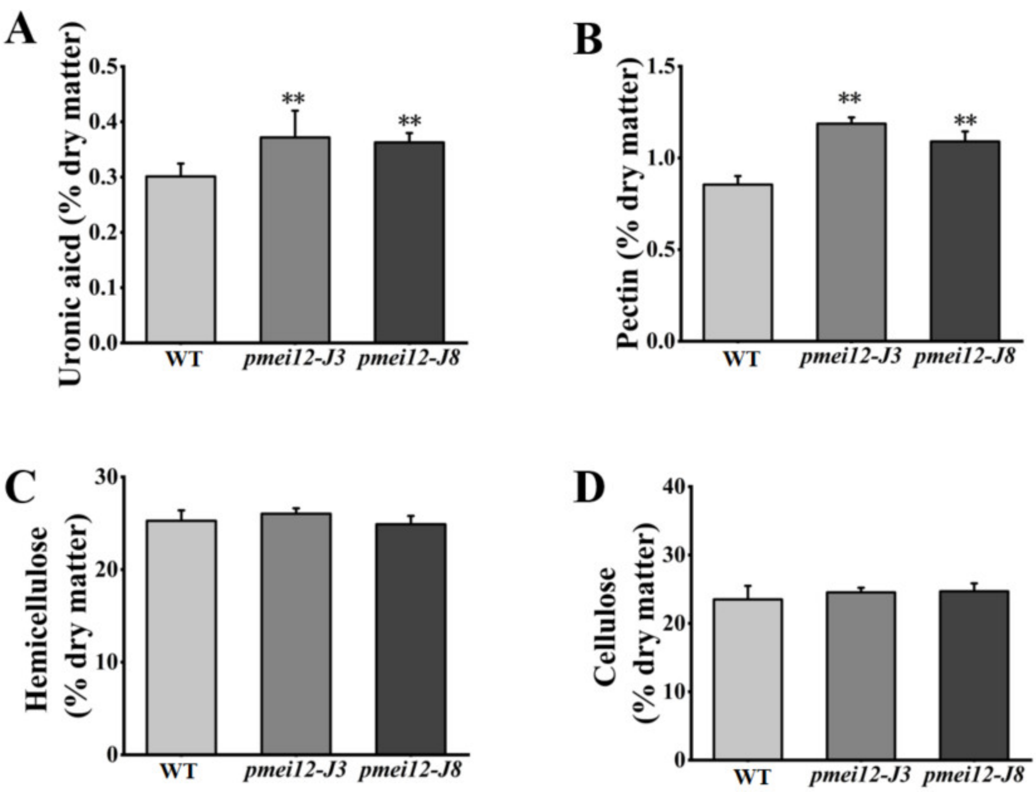

Figure S1. Determination of cell wall composition at mature stages of WT and pmei12s edited lines. (A) Uronic acid (Dry matter); (B) Pectin(Dry matter); (C) Hemicellulose (Dry matter); (D) Cellulose (Dry matter). * and * * respectively indicated the significant difference between pmei12 lines and WT by t-test as $P < 0.05$ and 0.01, n=3.

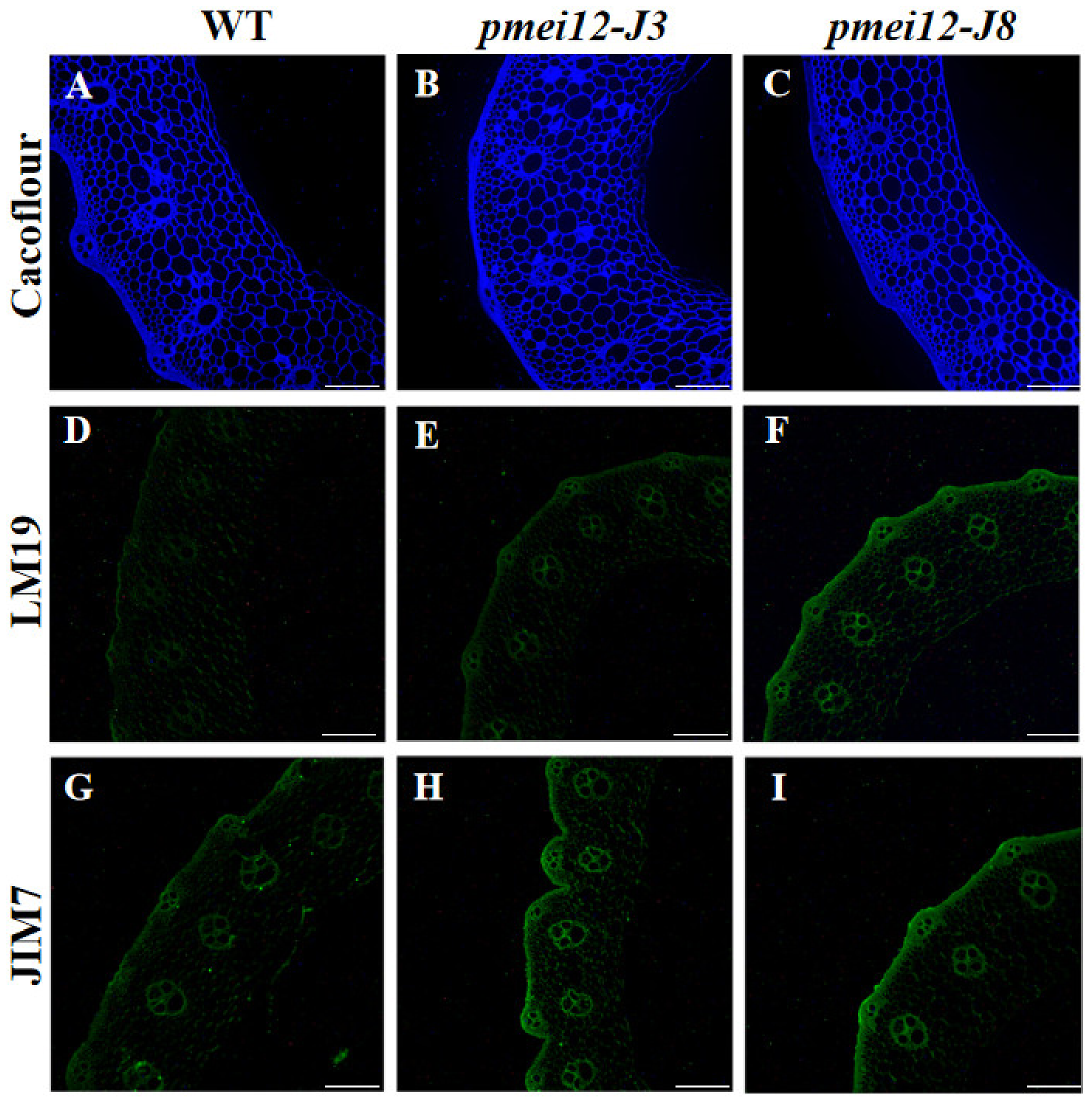

Figure S2. Immunofluorescence observation represents the content of pectin and uronic acid with low methyl ester (LM19) and high methyl ester (JIM7)(A-C). Staining cellulose with Cacofluor fluorescent whitening agent; (D-F) Monoclonal antibody LM19 specifically labeled the antigen epitope of low methyl HG; (G-I) Monoclonal antibody JIM7 specifically labeled the antigenic epitope of high methyl HG.

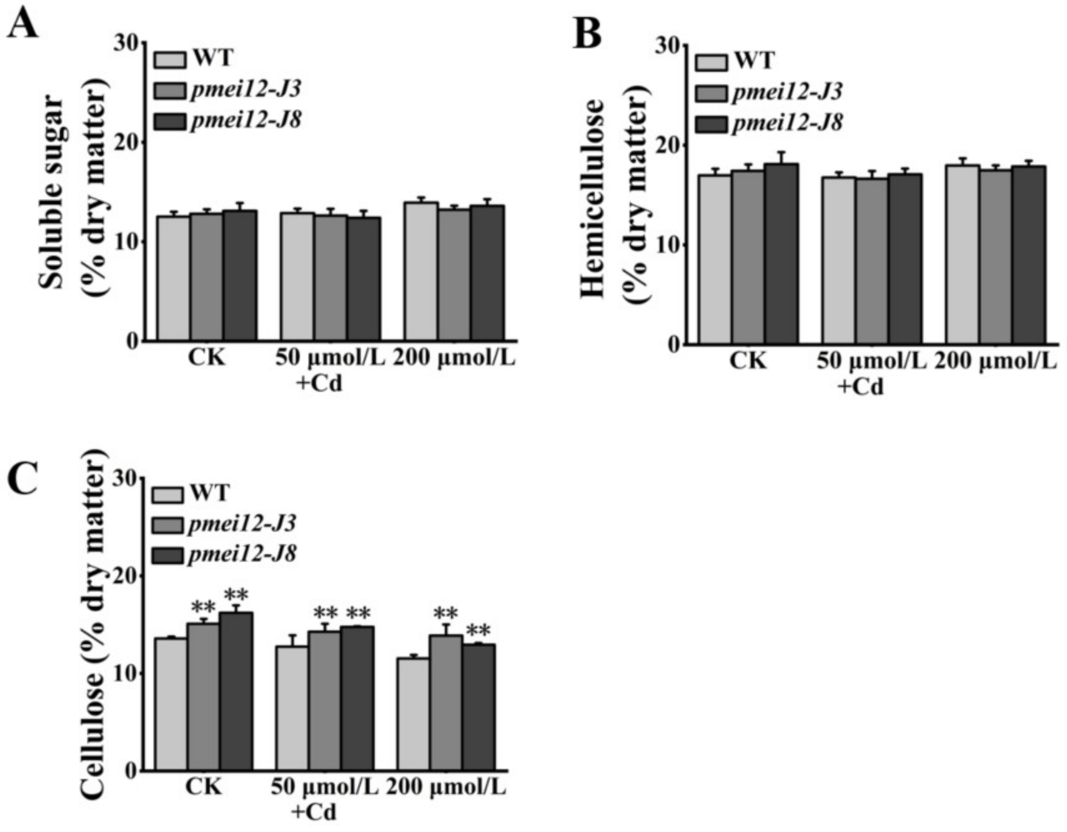

Figure S3. Seedling growth 21d was treated by heavy metal Cd stress. (A) Soluble sugar content in seeding (Dry matter); (B) Hemicellulose content in seeding (Dry matter); (C) Cellouse content in seeding (Dry matter). * and * * respectively indicated the significant difference between *pmei12* lines and WT by t-test as P < 0.05 and 0.01, Scale bars, (A) 5 cm, n=3.

1. Primer design for subcellular localization

Downloaded the full-length *OsPMEI12* gene sequence according to the TIGR website (http://rice.uga.edu/downloads_gad.shtml), and designed primers in the 5' and 3' regions of the UTR region of the gene sequence by using the primer 5 software. The position and restriction site (F:KpnI, R:BamHI) need to allow the gene to be transcribed normally.

2. Further details for hemicellulose and cellulose extraction

Extract alkali-soluble hemicellulose with 4 mol/L KOH: add 5.0 mL of 4 mol/L KOH (containing 1 mg/ml NaHB4) to the precipitate, and shake at 150 r/min for 1 h in a shaker at 25 °C. Centrifuge and collect supernatant. The precipitate was washed once with 5.0 mL of 4 mol/L KOH and twice with 5 mL of dH2O. All supernatants were collected and made up to volume and reading were determined as explained above by spectrophhotmeter (pentoses and hexoses was used as standards).

Extract total cellulose with sulfuric acid, and alkali-insoluble hemicellulose: add 3.0 ml of 67 % v/v) H2SO4 to the 4 mol/L KOH treatment residue, and mix well. 25 °C, 150 r/min, shaking for 1 h. Dilute the volume to 10.0 ml with dH2O distilled water, after cooling, centrifuge at 4000 r/min for 5 min, collect all the supernatants and take samples.

Colorimetric determination of Pentoses, Hexoses. The data of Hexoses measured in this step is total cellulose, Pentoses is alkali-insoluble hemicellulose, and total hemicellulose is the sum of alkali-soluble hemicellulose and alkali-insoluble hemicellulose.

2.2.6 Colorimetric determination of pentose, hexose and uronic acid

1. Determination of hexose content

1) Take an appropriate volume of sample into a 10 mL stoppered glass test tube, add dH2O to make up the total volume to 1.0 mL.

2) Slowly add 2.0 mL of 0.20% (w/v) anthrone sulfate reagent in a cold water bath, and shake quickly.

3) Heating in boiling water for 5 min and cooling to room temperature in tap

water.

4) Use a visible light spectrophotometer to measure the absorbance at a wavelength of 620 nm.

5) Use glucose as the standard to make a standard curve for the determination of hexose content:

6) Take 2.0 mL, 4.0 mL, 6.0 mL, 8.0 mL, 10.0 mL of 1.00 mg/mL glucose standard solution into a 100 mL volumetric flask, add water to the volume, and then take 1.0 mL of each of the above solutions into a 10 mL stoppered glass test tube , simultaneously measure the absorbance at a wavelength of 620 nm according to the above steps. 1.0 mL of dH2O was used as blank sample.

2. Determination of pentose content

1) Take an appropriate volume of sample into a 10 mL stoppered glass test tube, add dH2O to make up the total volume to 1.0 mL.

2) Add 134.0 μL Reagent A (6.00 g orcinol dissolved in 100.0 mL absolute ethanol).

3) Add 2.0 mL of Reagent B (0.100 g FeCl3 dissolved in 100.0 mL concentrated hydrochloric acid) and shake well.

4) Heating in boiling water for 20 min and cooling to room temperature in tap water.

5) Use a visible light spectrophotometer to measure the absorbance at a wavelength of 660 nm.

6) Use xylose as a standard to make a standard curve for the determination of hexose content:

7) Take 0.5 mL, 1.0 mL, 2.0 mL, 3.0 mL, 4.0 mL of 1.00 mg/mL xylose standard solution into a 100 mL volumetric flask, add water to the volume, and then take 1.0 mL of each of the above solutions into a 10 mL glass stopper In the test tube, the absorbance at the wavelength of 660 nm was measured simultaneously

according to the above experimental steps. 1.0 mL of dH2O was used as blank sample.

3. Determination of uronic acid content

1) Take an appropriate volume of sample into a 10 mL stoppered glass test tube, add dH2O to make up the total volume to 1.0 mL.

2) Add 5.0 mL of 0.50 % (w/v) sodium tetraborate/sulfuric acid solution and shake well.

3) Heating in boiling water for 5 min and cooling to room temperature in tap water.

4) Use a visible light spectrophotometer to measure the absorbance A at a wavelength of 520 nm, and recover the sample.

5) Add 100.0 μL of 0.15% (w/v) m-hydroxybiphenyl solution. After thorough mixing, let stand for 10 min. The absorbance B at a wavelength of 520 nm was measured again.

6) Use galacturonic acid as the standard to make a standard curve for the determination of hexose content:

7) Take 2.0 mL, 4.0 mL, 6.0 mL, 8.0 mL, 10.0 mL of 1.00 mg/mL galacturonic acid standard solution in a 100 mL volumetric flask, add water to the volume, and then take 1.0 mL of each of the above solutions into a 10 mL container. In a stoppered glass test tube, the absorbance at a wavelength of 520 nm was measured twice simultaneously according to the above experimental steps. 1.0 mL of dH2O was used as blank sample.

3. Preparation of Alcohol Insoluble Matter (AIR)

1. Weigh a fresh sample of about 10 g;
2. Grind it with liquid nitrogen;
3. Add 40 mL of chloroform-methanol (1:1, v/v), and place it on a shaker for 1 h

at room temperature for 150 r;

4. After removing the supernatant by centrifugation, add 40 mL of 70% ethanol to resuspend 4 times;

5. Finally, use 100% acetone to resuspend once, and put it in the fume hood to dry.

2.2.8 Determination of pectin methyl ester degree

1. Extract pectin in AIR;

2. Take 2 mL of pectin extract, centrifuge at 12000 r/min with a high-speed centrifuge, and remove the supernatant;

3. Resuspend in 2 mL of single-distilled water, take 0.2 mL of pectin extract, add 1 mL of 100 mM potassium phosphate buffer pH=8.0 to extract for 1 hour, and centrifuge at 16,000 g at 4 °C for 10 min.

4. Aspirate 200 μL of supernatant, add 90 μL of 0.2 M phosphate buffer pH=7 and 10 mL (0.01 U/mL alcohol oxidase in 0.2 M phosphate buffer), and incubate at 30 °C for 30 min.

5. 1 mg Purpald was dissolved in 100 uL of 0.5 M NaOH, added to the reaction system, vortexed vigorously, and incubated at 30 °C for 30 min.

6. Add 600 μL ddH20 and measure the absorbance at 550 nm.

4. Extraction of rice genomic DNA by CTAB method

1. Put about 2 cm of tender rice leaves into a 1.5 mL centrifuge tube, and grind with liquid nitrogen to fully disrupt the cells;

2. Add 650 μL of 1.5×CTAB and mix well with a toothpick;

3. 65 °C water bath for 30 min, shaking every 5-10 min;

4. Take out the centrifuge tube, put it at room temperature for 10 minutes, and add an equal volume of 650 μL of chloroform;

5. Mix well and shake for 15 minutes. Place at room temperature for 2 min,

12000 r/min, and centrifuge for 10 min;

6. Transfer 400 μL of supernatant to a new centrifuge tube and add an equal volume of isopropanol;

7. Shake for 3 minutes, place at room temperature for 2 minutes, and centrifuge at 12,000 r/min for 8 minutes;

8. Discard the supernatant, add 800 μL of 75% ethanol, invert 3 times, repeat once;

9. Centrifuge at 7500 r/min for 2 min, discard the supernatant, fully remove 75% ethanol with a 10 μL pipette, and air dry;

10. Add 50 μL ddH2O to dissolve and store at -20 °C.

5. Treatment methods of cadmium stress

Materials: Each material is planted in half a pot, and two groups of materials are planted in a total of four pots, two pots each.

1. Take photos of the growth of each experimental sample at 21 d (before cadmium application) and 30 d (harvest period) of seedling growth. Take 3 representative plants from each sample to measure their plant height, root length and number of roots. The leaf color was observed and recorded, and the tolerance or sensitivity of the gene editing line to the heavy metal cadmium was judged according to the morphological indicators of the growth of each sample during the harvest period.
